# Grandmothers’ mental health is associated with grandchildren’s emotional and behavioral development: a three-generation prospective study in Brazil

**DOI:** 10.1186/s12888-019-2166-8

**Published:** 2019-06-17

**Authors:** R. M. Pearson, I. Culpin, C. Loret de Mola, A. Matijasevich, I. S. Santos, B. L. Horta, F. C. Barros, A. Stein

**Affiliations:** 1 Centre for Academic Mental Health, Population Health Sciences, Bristol Medical School, University of Bristol, Oakfield House, Bristol, BS8 2BN UK; 2 NIHR Biomedical Research Centre, University of Bristol, Bristol, UK; 3 Postgraduate Program in Epidemiology, Federal University of Pelotas, Pelotas, Rio Grande do Sul Brazil; 4 Nursing Department, Federal University of Pelotas, Pelotas, Rio Grande do Sul Brazil; 5 Departamento de Medicina Preventiva, Faculdade de Medicina FMUSP, Universidade de São Paulo, São Paulo, SP Brazil; 6 Department of Psychiatry, Medical Sciences Division, University of Oxford, Oxford, UK; 7 MRC/Wits Rural Public Health and Health Transitions Research Unit (Agincourt), School of Public Health, Faculty of Health Sciences, University of the Witwatersrand, Johannesburg, South Africa

**Keywords:** Population-based study, Intergenerational, Depression, Anxiety, Grandparents, Emotional and behavioural problems, Pelotas Brazil

## Abstract

**Background:**

Maternal mental health is associated with an increased risk of emotional and behavioural problems in children, and the risk is partly explained by the negative impact of maternal depression on caregiving. The role of mental health in other family members, who in many contexts also provide substantial caregiving, has received far less attention. We examined the impact of grandmothers’ emotional symptoms, whose role in child care is increasing across the world, on internalizing and externalizing symptoms in grandchildren from a three-generation birth cohort study.

**Methods:**

Prospective data from three generations in two birth cohorts 22 years apart (1982 and 2004) in Pelotas, Brazil, were used (*n* = 92). Mental health in grandmothers and parents was assessed using the Self-Reported Questionnaire (SRQ-20). Grandchildren were members of the 2004 birth cohort, and behavioural and emotional problems were measured using the Child-Behaviour Checklist (CBCL) at age 4 years.

**Results:**

Grandmothers’ symptoms were associated with more emotional and behavioural problems in grandchildren after adjustment for confounding factors. The size of the associations between grandmothers’ and grandchildren mental health symptoms was comparable to the associations between maternal emotional symptoms and children emotional and behavioural problems. There was no evidence for associations with paternal symptoms. These effects were substantially stronger for maternal compared to paternal grandmothers.

**Conclusions:**

In some contexts, grandmothers’ mental health may be as important to grandchild emotional and behavioural development as maternal mental health. Interventions to improve the mental health of grandmothers, as well as parents, may be important to child mental health.

**Electronic supplementary material:**

The online version of this article (10.1186/s12888-019-2166-8) contains supplementary material, which is available to authorized users.

## Background

It is well-established that *maternal* depression is associated with a broad range of emotional and behavioral disturbances in children, and that this risk is partially explained by the negative impact of depression on parent-child interactions [1]. Indeed, interventions that improve depressed mother-infant interactions have shown positive effects on the child [2]. Depression is likely to have the same impact on caregiving, irrespective of who cares for the child. However, the impact of depression on other key care-givers and its impact on child development has received little empirical attention. These insights are important to inform which family members should be included in interventions aimed at improving emotional and behavioral development of children. Evidence suggests that in Western cultures, where fathers may adopt a greater role in child-rearing, paternal depression has adverse impact on child development [3]. There is also evidence to suggest the importance of grandparents’ mental health for child development, however, the findings are inconsistent, and the effects may vary depending on the cultural context [4–7].

For instance, in many non-Western cultures, such as Latin America, grandmothers are culturally designated advisors on child-rearing as well as active caregivers [8]. Qualitative evidence suggests that, in these contexts, grandmothers are highly valued with fathers having relatively limited influence during early infancy [8]. Given the rising number of mothers in full-time work in both non- and Western world [9], grandmothers are increasingly taking on the role of preschool day-carers. For instance, in the US and Europe over half of working mothers rely on relatives for childcare, most frequently *maternal* grandmothers, who also provide emotional and financial support to parents [10]. Thus, grandmothers’ mental health may impact grandchildren directly, through frequent caregiving, and indirectly, through influencing their parents [7]. This pathway may be particularly relevant for maternal grandmothers given recent evidence suggesting that maternal depression predicts daughter’s (but not son’s) depression in adulthood [11].

As well as any effects of the grandmothers’ depression on child rearing and mothers’ mental health, associations across generations could also be explained by genetic inheritance of vulnerability, even if the grandmother had no contact with the parent or the grandchild. The genetic heritability of depression, however, is relatively low (up to 37%) [12] and this would become further diluted across generations. This means that it will only contribute to some of the association. In addition, if explained by genetic factors alone, intergenerational associations from maternal and paternal lines would be expected to be the same, given that the genetic contribution is equivalent for maternal and paternal grandparents. In contrast, the child-rearing contribution from maternal and paternal grandmothers are likely to differ. Environmental characteristics that are associated with mental health, such as poverty, are often shared across generations. However, this would also be expected to be at least as strong for paternal as for maternal grandmothers, particularly as family income is more often determined by fathers. In summary, there are several reasons to believe that grandmother’s (especially maternal grandmothers) mental health is important to their grandchildren’s development, with environmental contributions playing an important role.

Longitudinal evidence in high-income countries (HIC) suggests that parent-reported history of grandparent depression/anxiety is associated with increased risk of such disorders in grandchildren [4–6, 12]. However, the retrospective nature of parental reports is likely to be biased by parental mental health as parents may not be aware of the emotional state of their own parents, especially if the symptoms are mild. Parents are more likely to be aware of mental health problems in their own parents if they also suffer from mental health problems. Indeed, some studies only find associations with maternal, and not paternal reports of grandparent mental health, highlighting the influence of the reporter [4]. Thus, studies using prospectively collected measures of symptoms reported directly by the grandparents are important and presently lacking.

To the best of our knowledge, only two studies up-to-date have examined grandparents’ mental health prospectively and found evidence of intergenerational associations between mental health disorders [7, 13]. One of these studies focused on major depressive disorders with cases being selected from outpatient specialty services, which is a selective group with severe depression presentation [13]. However, the majority of depression is treated in primary care with mild symptoms being common at a population level and a cause of significant economic and health burden [14]. Recall of milder symptoms by family members may be particularly affected by recall bias as they are often not expressed by those who are affected. This highlights the need for population-based prospective studies to address this limitation. One population-based study of grandparent reported (grandmothers only) mental health and grandchild emotional and behavioural development using the UK 1970 British Cohort Study data found evidence of an association between grandmother symptoms of depression and anxiety and grandchild emotional and behavioural problems. [7]. However, the relationship was reported to operate indirectly through parental mental health. Furthemore, there are no investigations spanning three generations using prospective measures of symptoms in population samples in each generation from the Latin America, where grandparents play a substantial role in childcare. We used prospectively collected data from the Pelotas study, a large birth cohort based in the south of Brazil, to address some of these gaps in the literature. Our research questions were:

1. Are grandmothers’ emotional symptoms associated with behavioral and emotional problems in grandchildren?
2. Are these association stronger in maternal, compared to paternal grandmothers, reflecting the relatively greater role of maternal grandmothers in childcare?

## Method

### Study participants and procedures

**Fig. 1:**
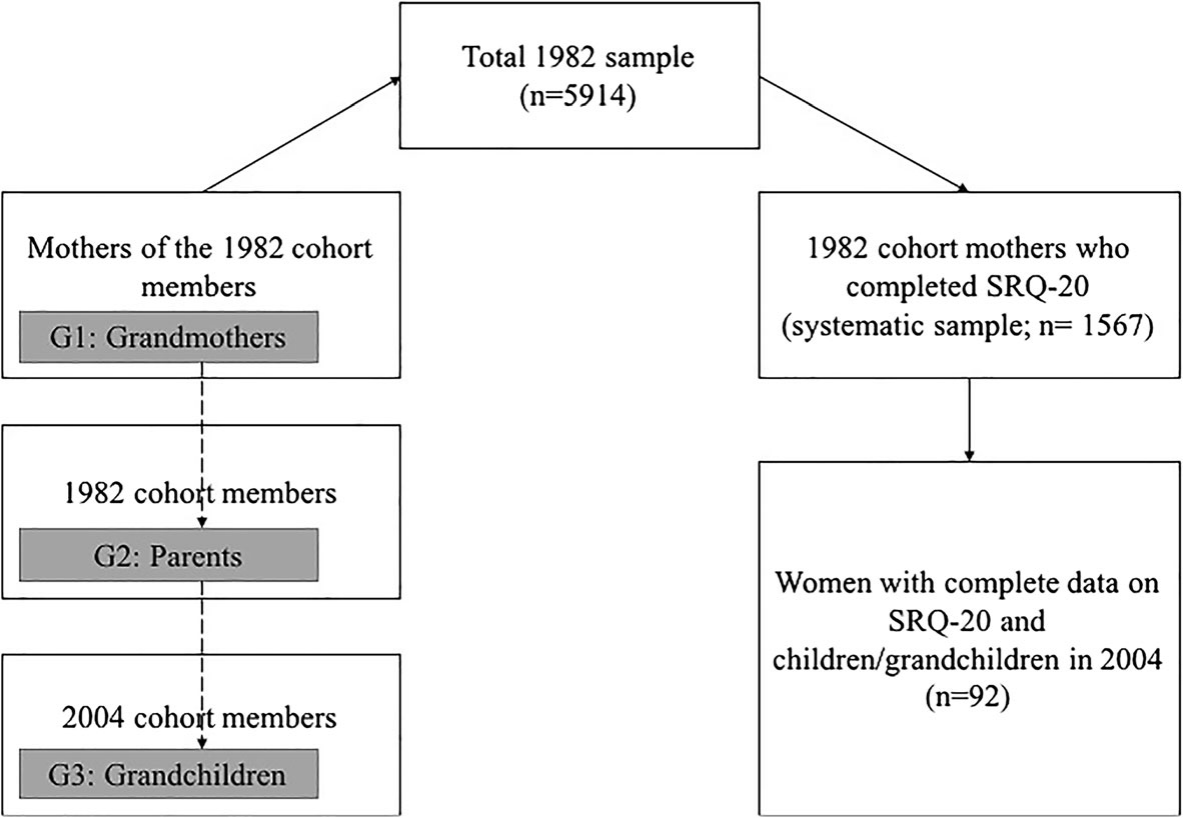
Illustration of the three-generation linkage

The study combined data from two birth cohorts in Pelotas, a southern Brazilian city with a population of 328,000 inhabitants (2010 Brazilian population census). Generation 2 (G2 referred to as parents) are members of the 1982 birth-cohort; Generation 1 (G1 referred to as grandmothers) are the mothers of the 1982 birth-cohort; and Generation 3 (G3 referred to as grandchildren) are members of the 2004 cohort with parents who are in the 1982 cohort (Fig. 1). Common mental health disorders (CMDs), especially depression and anxiety, were assessed using the same instruments in parents and grandmothers (Self-Reported Questionnaire; SRQ-20), whilst emotional and behavioural problems in grandchildren were measured at age 4 years.

#### 1982 Cohort

During the whole of 1982, the three maternity hospitals were visited daily, and 7392 births were recorded [15]. Of these, 6011 infants were born to mothers living in the urban area of Pelotas. Using data from birth registration and from a city census, another 46 children, who were delivered at home in 1982 were identified, so that the hospital sample accounted for 99.2% of all births in the city (the refusal rate to participate was < 1%) [15]. The starting sample of the 1982 Cohort was 5914 mothers (grandmothers in this analysis), who were followed up on several occasions. Detailed methodology describing the follow-up is provided in further cohort profile papers [15, 16]. In 2001, 70 census tracts (defined regions) were systematically selected (27% of the total due to funding constraints) and all households in those tracts were visited. This led to 72% of the cohort members expected to be living in those tracts to be traced [15]. Male and female cohort members were identified and their mothers (grandmothers in this analysis) completed the self-reported questionnaire (SRQ-20) validated for Brazil [16, 17]. Thus, the grandmother sample represents this systematic sample. In 2003–2004, all cohort members (parents in this analysis) were invited to undergo home assessment, including the SRQ-20, using information from existing addresses and intense mass media campaign.

#### 2004-cohort

The 2004 Pelotas cohort consisted of a population of women with children born in 2004 in Pelotas [18]. All mothers who resided in the urban area of Pelotas, or in the adjacent neighborhood of Jardim América (part of the municipality of Capão do Leão), were approached to participate and interviewed within 24-h of delivery [19]. Less than 1% of mothers refused, given a total of 4231 newborns in the study were successfully recruited to the study [18, 19]. All cohort children were followed up at the age of 4 years old (Mean = 49.5 months, SD = 1.7).

The Ethical Review Board of the Faculty of Medicine of the Federal University of Pelotas approved the 1982 and 2004 studies. Written informed consent was obtained at each follow-up from mothers or directly from participants when they were > 18 years old. In 1982, verbal consent was obtained from the mothers following the standard practice at that time.

### Measures

#### Exposure measures (G1 and G2): self-reported questionnaire (SRQ-20)

The SRQ-20 test consists of 20 items pertaining to physical and psychological symptoms of depression and anxiety during 30 days prior to the interview [17]. The SRQ-20 has good sensitivity (83%) and specificity (80%) for detecting depressive or anxiety disorders against a psychiatric interview [17]. In order to maximise statistical power, we used continuous scores in the analyses. SRQ-20 was measured in a systematic sub-sample of grandmothers (G1) when parents (G2) were aged 18/19 years old in 2001, thus, 3 years before the birth of their index grandchild (G3). The SRQ-20 was also administered to 2004 cohort members (parents for this analysis) aged 22/23 years old as part of the 1982 cohort assessment. For those cohort members with children in the 2004 cohort this timing represents the postnatal year of their child (G3 grandchildren). The items according to specific symptoms clusters are as follows: *depressed mood* (e.g., ‘do you feel unhappy?’, ‘have you lost interest in things?’, ‘do you cry more than usual?’, ‘do you enjoy your daily activities?’); *anxiety* (‘are you easily frightened?’, ‘do you feel nervous, tense or worried?’, ‘do your hands tremor?’); *depressed thoughts/hopelessness* (‘do you feel a worthless person?’, ‘has the thought of ending your life been in your mind?’, ‘are you unable to play a useful part in life?’); *poor concentration* (‘do you have trouble thinking clearly?’, ‘do you find it difficult to make decisions?’, ‘is your daily work suffering?’); *somatic* (‘do you often have a headache?’, ‘is your appetite poor?’, ‘is your digestion poor?’, ‘do you have uncomfortable feelings in your stomach?’) and *sleep/fatigue* (‘do you sleep badly?’, ‘do you feel tired all the time?’, ‘are you easily tired?’).

#### Outcome measures in grandchildren (G3): Child Behavior Checklist (CBCL)

Child emotional and behavioural problems were assessed at age 4 years using the Child Behavior Checklist (CBCL) [20]. We used the 4–18-year version, the only version validated in Brazil [20, 21]. The CBCL contains 118 behavioral and emotional items, which were scored by mothers. A profile of childhood psychological problems provided scores on eight empirically derived scales: withdrawn, somatic complaints, anxious/depressed, social problems, thought problems, attention problems, aggressive behavior, and rule-breaking behavior. Data from these scales were summed to provide an overall continuous score (total emotional and behavioural problems) and were also grouped into two broad dimensions: internalizing and externalizing symptoms [22].

#### Confounding variables

Parental and socioeconomic characteristics identified in previous studies as potential confounding variables were: maternal (grandmothers in this analysis) schooling (highest grade of schooling successfully completed); household assets index (derived using factor analysis and based on the ownership of household goods); sex of parent (G2) and grandchild (G3); age at time of assessment for all generations; parity of all generations; G1 and G2 skin color as assessed by the field workers (proxy for the ancestral background, because miscegenation in Brazil is highly prevalent); and marital status (G2), which may influence the support needed from the grandmothers.

### Statistical analysis

#### Statistical power

There were 92 grandchildren (G3) with complete grandmother (G1) and parental (G2) data. With this sample size, the study has 78% power to detect a correlation of 0.25 between grandmothers (G1) and grandchildren (G3), which corresponds to the correlation previously found in the 1982 cohort between grandmothers (G1) and parents (G2) in adulthood. Thus, we had sufficient power to detect a correlation of a similar magnitude. Missing data was mostly due to only a systematic sample of grandmothers completing the SRQ-20 in 2001. Given that the sample did not differ on socioeconomic or psychological indices (i.e. systematic sample), any bias associated with missing data is likely to be minimal. All members of 1982 cohort were invited to complete the SRQ-20 in 2004, thus, we had a greater sample size (*n* = 231; 90 fathers and 141 mothers) with complete grandchild (G3) and parental (G2) data. Our study sample comprised women (G1) with complete data on SRQ-20 and children(G2)/grandchildren (G3) in 2004 (*n* = 92; Fig. 1). This sample size was used to provide more precise estimates for G2-G3 associations presented in the Additional file 1).

#### Primary analysis

First, the association between grandmother (G1) SRQ-20 and grandchild (G3) CBCL total emotional and behavioural scores was examined using linear regression analysis unadjusted and adjusted for parental and socioeconomic confounders. Second, we compared these associations with the associations between parents (G2) and grandchildren (G3) across the complete case sample. In a separate step, we also mutually adjusted grandmother (G2) and parent (G3) associations for each other (i.e, grandmother and mother SRQ-20 scores were included into the same model. All analyses were conducted in Stata v.13 (StataCorp., USA).

#### Path analysis

We estimated the proportion of any association between grandmother (G1) SRQ-20 and grandchild (G3) CBCL that is explained by parental (G2) SRQ-20 symptoms using path analysis in Mplus v.7. The product of coefficients strategy was used to estimate indirect effects [23]. Standardised path coefficients and standard errors were estimated using bootstrapping methodology (5000 models) using a maximum likelihood (ML) robust estimator. Bootstrapping is a non-parametric test, thus, it does not rely on the assumptions of normality that are often not met when calculating indirect effects. The path analyses was likely to be underpowered to detect mediated effects.

## Results

### Sample

As a sample of relatively young parental age, families included in the three-generation sample (*n* = 92) were more likely to have low income as compared to the 1982 or 2004 cohorts as a whole, reflecting earlier childbearing among the poor (Table 1). Parents (G2) were also more likely to be primiparous. However, parents (G2) were not more likely to be unmarried or to have a less involved father or to be employed. Importantly, grandmothers included in the 3 generation analyses were not more likely to score above the threshold on the SRQ-20 than the rest of the mothers in 1982 sample. The mean emotional problem score in the grandchildren was 6.9 (SD 4.8) and behavioral problem score was 16.9 (SD 7.5). Mean SRQ score in parents was 5.5 (SD 4.4) in mothers, 4.5 (SD 4.2) in fathers and in grandparents, 6.3 (SD 4.6) in paternal grandmothers and 7.1 (SD 4.3) in maternal grandmothers.

**Table 1:** Comparison of the three-generation sample and 1982 and 2004 Cohorts

Sample characteristics	Three-generational sample (*n* = 92 with G1, G2)	Whole 1982 sample (*n* = 5914)	Whole 2004 sample (*n* = 4231)	Chi2, p across groups
% Income in minimum wages				
*< 1*	31%	22%	21%	11.8, 0.019
*1.1–3*	55%	47%	46%	
*> 3*	14%	31%	33%	
Grandmother age at birth of G2				
*< 20*	17%	15%	N/A	0.20, 0.904
*20–29*	58%	58%		
*+ 30*	25%	27%		
Marital status of parent (G2)				
*% Married*	82%	N/A	83%	0.034, 0.852
Parity of G2 mother				
*% Primiparous*	56%	N/A	39%	5.8, 0.016
Involvement of G2 father				
*% Play with child*	85%	N/A	87%	0.16, 0.684
*% Put child to bed*	57%		55%	0.89, 0.344
*% Bathe child*	39%		35%	0.34, 0.558
*% Educate child*	81%		80%	0.03, 0.858
% Grandmothers (G1) above SRQ-20^a^ threshold	28%	27%	N/A	0.16, 0.692
% Mothers (G2) work when child (G3) is 48 months	68%	N/A	65%	0.20, 0.653
% Grandmothers (G1) work when mother (G2) was 2 years old	35%	N/A	34%	0.02, 0.882

*Note:* ^a^ SRQ-20: Self-Reported Questionnaire

#### Association between generations and emotional and behavioural problems in grandchildren

There was strong evidence that maternal grandmothers’ mental health was associated with increased emotional [adjusted β-coefficient: 2.1, 95% CI 0.8, 3.4, *p* = 0.001] and behavioural [adjusted β-coefficient: 2.5, 95% CI 0.5, 4.7, *p* = 0.018] problems in grandchildren in the unadjusted and adjusted models (Tables 2 and 3). There was no evidence that any of the individual confounding variables had an influence on the associations. The effect sizes were comparable to the associations observed between parental symptoms and grandchild emotional and behavioural problems. There was evidence for an interaction between parental gender and grandmother symptoms, with associations being clearly limited to maternal grandmothers. This echoed the parental gender by parent symptom interaction, where associations between parental SRQ-20 scores and grandchild problems were limited to mothers. Results for the G2 to G3 associations using all available data were similar are presented in Additional file 1.

**Table 2:** Linear regression investigating associations between SRQ-20 scores and grandchild emotional problems across generations

	Model 1: Unadjusted β-coefficients (95% CI, p) for a 5-point increase in SRQ-20^d^ score			Model 2: Adjusted^a^ β-coefficients (95% CI, p) for a 5-point increase in SRQ-20^d^ score		
G1 to G3 (*n* = 92)	All grandmothers	Maternal grandmothers (*n* = 68)	Paternal grandmothers (*n* = 24)	All grandmothers^b^	Maternal grandmothers (*n* = 68)	Paternal grandmothers (*n* = 24)
Emotional problems in G3	1.3 (0.2, 2.4), 0.021	2.0 (0.8, 3.0), 0.001	− 0.1 (−3.0, 2.7), 0.934	1.3 (0.1, 2.6), 0.037	2.1 (0.8, 3.4), 0.001	− 0.9 (−5.4, 3.5), 0.666
G2 to G3 (*n* = 92)	All parents	Mothers	Fathers	All parents^c^	Mothers	Fathers
Emotional problems in G3	0.9 (−16, 2.1), 0.092	1.7 (0.6, 2.8), 0.003	−1.2 (−4.3, 1.9), 0.446	0.9 (−26, 2.2), 0.123	1.8 (0.6, 3.0), 0.005	− 1.2 (− 5.8, 3.4), 0.578

*Note*: ^d^SRQ-20: Self-Reported Questionnaire; ^a^Adjusted for grandmother and parent schooling, family income, grandmother age, gender, grandmother ethnicity, grandchild age at assessment, parental and grandparental skin colour and gender, parental parity and marital status; ^b^Parent gender x grandmother symptoms interaction in Model 2: *p* = 0.049; ^c^Parent gender x symptoms interaction in Model 2: *p* = 0.033

**Table 3:** Linear regression investigating associations between SRQ-20 scores and grandchild behavioural problems across generations

	Model 1: Unadjusted β-coefficients (95% CI, p) for a 5-point increase in SRQ-20^d^ score			Model 2: Adjusted^a^ β-coefficients (95% CI, p) for a 5-point increase in SRQ-20^d^ score		
G1 to G3 (*n* = 92)	All grandmothers	Maternal grandmothers (*n* = 68)	Paternal grandmothers (*n* = 24)	All grandmothers^b^	Maternal grandmothers (*n* = 68)	Paternal grandmothers (*n* = 24)
Behavioural problems in G3	1.8 (0.1, 3.6), 0.042	2.7 (0.7, 4.6), 0.008	−0.2 (−4.2, 4.6), 0.912	1.7 (− 0.3, 3.7), 0.94	2.5 (0.5, 4.7), 0.018	−3.4 (−11.5, 4.7), 0.377
G2 to G3 (*n* = 92)	All parents	Mothers	Fathers	All parents^c^	Mothers	Fathers
Behavioural problems in G3	1.7 (−0.2, 3.7), 0.094	2.7 (0.7, 4.6), 0.008	−0.2 (−4.19, 3.8), 0.912	0.84 (− 0.9, 2.7), 0.366	2.0 (0.4, 3.9), 0.046	−2.7 (−8.9, 3.4), 0.355

*Note*: ^d^SRQ-20: Self-Reported Questionnaire; ^a^Adjusted for grandmother and parent schooling, family income, grandmother age, gender, grandmother ethnicity, grandchild age at assessment, parental and grandparental skin colour and gender, parental parity and marital status; ^b^Parent gender x grandmother symptoms interaction in Model 2: *p* = 0.042; ^c^Parent x gender symptoms interaction in Model 2: p = 0.018

#### Mediation analyses

**Fig. 2:**
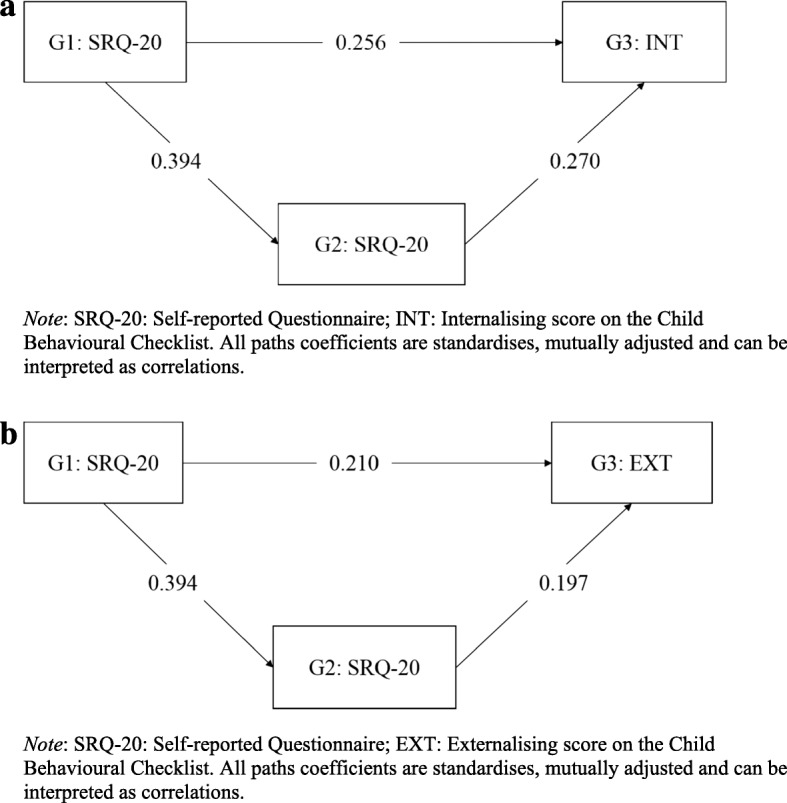
**a** Mediation through maternal symptoms for internalising symptoms. **b** Mediation through maternal symptoms for externalising symptoms

The mutually adjusted linear regression models suggested that both associations attenuate when co-adjusted, indicating some shared effects (Table 4). However, there was remaining evidence for maternal grandmother in particular, which may indicate a more robust influence of grandmothers over mothers, although statistical power was reduced to detect such effects. There was evidence to suggest that a proportion of the associations between grandmother depression and grandchild emotional and behavioural problems may be mediated by maternal symptoms (Table 5 and Fig. 2a-b). However, a substantial proportion of the variance remained through a direct pathway.

**Table 4:** Mutually adjusted linear regression analyses for the associations between maternal and maternal grandmother mental health and grandchild emotional and behavioural problems (*n* = 68)

	Maternal grandmothers, adjusted for mothers’ SRQ-20^a^ β-coefficient (95% CI), p	Mothers, adjusted for maternal grandmothers’ SRQ-20 β-coefficient (95% CI), p
Emotional	1.6 (0.2, 3.0), 0.023	1.1 (−0.2, 2.4), 0.086
Behavioural	2.1 (−0.3, 4.5), 0.084	1.5 (−0.7, 3.8), 0.175

*Note*: ^a^SRQ-20: Self-Reported Questionnaire

**Table 5:** Total, direct and indirect effects of maternal grandmother SRQ-20 on grandchild emotional and behavioural problems^a^ (mothers and maternal grandmothers only; *n* = 68)

*n* = 68	Total	Indirect effect through maternal perinatal SRQ-20	Direct	Proportion of effect explained through indirect pathway
Emotional	0.363, p = 0.001	0.106, *p* = 0.082	0.256, *p* = 0.048	29%
Behavioural	0.287, *p* = 0.025	0.078, *p* = 0.135	0.210, *p* = 0.151	27%

*Note*: ^a^Standardised β-coefficients

## Discussion

To the best of our knowledge, this study is the first population-based three-generation study using prospectively collected measures of mental health from low- and middle-income country (LMIC) that examined the association between grandmothers’ mental health and grandchildren emotional and behavioural development. We found evidence that grandmothers’ emotional symptoms were associated with emotional and behavioral problems in their grandchildren. The size of these associations was comparable to the associations between maternal symptoms and emotional and behavioural problems in children. The grandmothers’ associations remained after accounting for the indirect pathways through the impact of grandmothers’ symptoms on the grandchild’s parent, suggesting that grandmothers may have a direct impact on their grandchildren development. This is in contrast to studies from the high-income countries (HIC), where the influence of the grandmothers’ mental health was fully explained by maternal mental health [5, 7]. This finding could suggest a stronger direct contribution of grandmothers in Brazil where the rate of female labor force participation has tripled even in comparison to the most South American countries [9].

Effects were considerably stronger for (and generally limited to) maternal rather than paternal grandmothers, with statistical evidence for the effect modification. This finding is most consistent with the greater role of maternal grandmothers in child-rearing, whereas genetic inheritance or shared environmental adversity (e.g., poverty) would seem to predict equivalent associations and no effect modification by parental gender. However, we cannot completely rule out the possibility of differential inheritance of genetic vulnerability from maternal versus paternal grandmothers through a more complex genetic mechanism (such as mitochondrial DNA). Currently, there is no evidence that this type of genetic mechanism is important for emotional or behavioural outcomes [24], however, this may change with further developments in genetic research.

There was no evidence that fathers’ depression had a negative impact on child emotional or behavioral outcomes. The sample size comprising fathers was relatively small, thus, there was limited power to detect small effect sizes. However, it is worth noting that regression coefficients for paternal effects were generally negative or minimal. In addition, this implies that any association would have been smaller than that of grandmothers for which the sample size was equivalent. This finding is consistent with existing evidence suggesting that in Latin America grandmothers play a greater role in early years childcare than fathers [8]. Grandmothers are perceived as culturally designated advisors on child-rearing practices as well as active caregivers, representing the ‘authority’ figure in families and reflecting cultural hierarchy and respect for age and experience, whilst fathers play a relatively limited role in day-to-day caregiving within the family system [8].

### Strengths and limitations

The strengths of the study include population-based design and the linkage of prospectively collected three-generational data from the same family. The same instrument was used to measure symptoms in both grandmothers and parents allowing direct comparisons of associations. In addition, data was available for both maternal and paternal grandmothers and for fathers and mothers. Importantly, given the linkage between existing cohorts, fathers were members of the 1982 cohort from birth rather than being recruited into the study as fathers. This may explain the differences between the current findings and previous research investigating effects of paternal depression on the child with fathers who took part as fathers of the study index child. Such selection of fathers may have resulted in the recruitment of a more engaged, and, thus, more influential group of fathers who are highly involved in bringing up their children.

The findings need to be interpreted in light of several limitations. Firstly, the sample with three-generational data was naturally selected according to a specific variable, i.e. parents (G-2) having a child in Pelotas during 2004 when they were 22 years old. This parental age is lower than the mean age of parents in the 2004 cohort, which is 26 years old. A Brazil-based study found that grandmothers’ involvement in day-to-day childcare was greatest for younger mothers [25]. In addition, to be included in the current study *both* parents (G-2) and grandchildren (G-3) must have been born in Pelotas. Thus, parents who moved in or out of Pelotas would not have been included. Parents who remained in the city in which they were born may have stronger and wider family support network than those who move away. Nonetheless, findings of the current study demonstrate that, in certain circumstances, grandmothers appear to be as important as mothers when it comes to the next generation’s mental health.

Secondly, the sample size was relatively small comprising those with complete SRQ-20 measures. It is important to note that the relatively small sample size was due to purposeful sampling and not loss to follow-up, suggesting that the sample was not biased. In addition, the power calculation suggested that the study was powered to detect associations of the magnitude previously reported because we were able to use continuous scores for all variables. It should be noted, however, that the path analysis was likely to be underpowered and should be interpreted as exploratory. There were particularly low numbers of paternal grandmothers and fathers, which may reflect higher average age of fathers compared to mothers with fewer males from the 1982 cohort becoming fathers by age 22 years. Thirdly, child emotional and behavioural problems were parent-reported, most commonly by the mother. This may lead to reporting biases, whereby parents with mental health problems tend to overestimate emotional and behavioural symptoms in their children. However, this would influence the associations with mothers’ mental health more so than grandmothers’, reducing the likelihood of biasing the associations between grandmothers’ and grandchildren mental health problems.

We did not have the data on the specific roles that grandmothers play, such as the type and frequency of child care, geographical proximity to their grandchildren, or their ‘authority’ status in the family. In addition, other cultural, social and geographical factors may affect the health and well-being of the family, thus, future research should focus on disentangling the complex mechanisms by which grandmothers’ mental health is related to the child development, particularly in light of these preliminary findings suggesting that grandmothers’ depression and anxiety have an important effect. Thorough examination of the effects of these factors was beyond the scope of this study, as they may be on a causal pathway between grandmothers’ and grandchildren mental health driving changes in the pattern of caregiving practices. Existing research from HIC has found that the frequency of contact with grandparents did not alter the association between grandparents’ and grandchildren mental health [4]. However, as noted previously, the grandparent to grandchild associations were found to operate mainly indirectly through parental mental health. Thus, the direct contact with grandparents may be less relevant in HIC compared to LMIC context, where maternal grandmothers may have a greater direct influence on their grandchildren through day-to-day involvement in child-rearing practices [8].

Other putative mechanisms are also possible. For instance, grandmothers often advise mothers on childcare practices, thus, grandmothers’ depression may influence the quality and quantity of such advice, which may, in turn, influence the child. It is also possible that grandmothers’ depression influences maternal reports of child mental health independently of the effects on maternal mental health. For instance, a grandmother who experiences mental health difficulties may be easily frustrated by the child exaggerating child’s emotional and behavioural difficulties, which, in turn, influences parental reports of such problems. In addition, depression in grandmothers, who are involved in day-to-day care of the grandchildren, may interfere with their ability to provide sensitive and consistent responses to the child [26]. This is consistent with evidence suggesting that depression in parents is associated with increased use of harsh parenting such as hitting and shouting at the child [26], thus, similar practices are likely in the context of grandparents’ depression. These negative care-giving practices are in turn associated with emotional and behavioral problems in children [1].

## Conclusions and implications

Our study provides support to the importance of grandmothers’ mental health for grandchild emotional and behavioural development; in some contexts, it is as important as maternal mental health and more so than paternal mental health. Thus, interventions to improve the mental health of grandmothers, as well as parents, may be important to child mental health. Current intervention strategies aimed at improving infant emotional and behavioral development often focus on the mother-infant dyad, and, increasingly, on the role of the father [1]. However, the role of other family members is given little attention, and the key influence of grandparents in non-Western cultures on the children has rarely been utilized in public health interventions [8]. Further research is needed to understand the circumstances under which the grandmothers’ mental health may be important for the grandchildren development. The current findings suggest that interventions, especially in Brazil, should consider grandmothers’, particularly maternal grandmothers’, mental health as well as that of parents as this may increase the potential to increase the effectiveness of intervention strategies and to improve child emotional and behavioral development.

## Additional file

Additional file 1: **Table S1.** Linear regression investigating associations between G2 and G3 for all available data giving more precise estimates. (DOCX 16 kb)

## Data Availability

The datasets generated and/or analysed during the current study are not publicly available due to confidentiality issues, however are available from the corresponding author on reasonable request.
