# Supplementary material for: Grandmothers’ mental health is associated with grandchildren’s emotional and behavioral development: a three-generation prospective study in Brazil
Source: BMC Psychiatry. 2019 Jun 17;19:184. doi: 10.1186/s12888-019-2166-8 (PMC6580571; doi:10.1186/s12888-019-2166-8)
Supplement: Supplementary file 1 — Table S1. Linear regression investigating associations between G2 and G3 for all available data giving more precise estimates. (DOCX 16 kb) [file 12888_2019_2166_MOESM1_ESM.docx]

**Additional file 1**

**Table S1.** Linear regression investigating associations between G2 and G3 for all available data giving more precise estimates

| Emotional problems in grandchildren | Model 1: Unadjusted β-coefficients (95% CI, p) for a 5-point increase in SRQ-20^*^ score | | | Model 2: Adjusted^a^ β-coefficients (95% CI, p) for a 5-point increase in SRQ-20^*^ score | | | Parent gender x parental symptoms interaction |
| --- | --- | --- | --- | --- | --- | --- | --- |
|  | All parents | Mothers  (n=141) | Fathers  (n=90) | All parents | Mothers  (n=141) | Fathers  (n=90) | p=0.005 |
| G2 to G3  (n=231) | 1.3 (0.5, 2.2), 0.002 | 2.0 (1.2, 3.2), <0.001 | -0.7 (-2.1, 0.8), 0.335 | 1.4 (0.5, 2.2), 0.002 | 2.2 (1.1, 3.3), <0.001 | -0.7 (-2.3, 0.8), 0.376 |  |
|  |  |  |  |  |  |  |  |
| Behavioural problems in grandchildren | All parents | Mothers  (n=141) | Fathers  (n=90) | All parents | Mothers  (n=141) | Fathers  (n=90) | p=0.018 |
| G2 to G3  (n=231) | 1.4 (0.3, 2.5), 0.011 | 2.3 (1.1, 3.6), <0.001 | -0.7 (-2.9, 1.5), 0.335 | 1.4 (0.2, 2.5), 0.017 | 2.3 (0.9, 3.6), <0.001 | -0.9 (-3.2, 1.4), 0.450 |  |

*Note*: ^*^SRQ-20: Self-Reported Questionnaire; ^a^Adjusted for grandmother and parent schooling, family income, grandmother age, gender, grandmother ethnicity, grandchild age at assessment, parental and grandparental skin colour and gender, parental parity and marital status.
